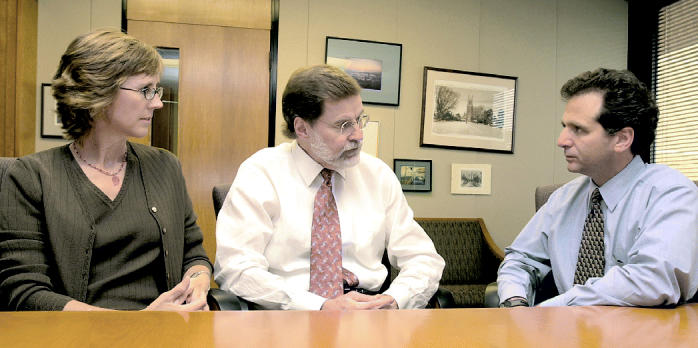# The Need for Exposure Health Sciences

**DOI:** 10.1289/ehp.113-1281298

**Published:** 2005-10

**Authors:** David A. Schwartz, Brenda Weis, Samuel H. Wilson

**Affiliations:** Director, NIEHS and NTP, E-mail: david.schwartz@niehs.nih.gov; Special Assistant to the Director, E-mail: weis@niehs.nih.gov; Deputy Director, NIEHS, E-mail: wilson5@niehs.nih.gov

Although potentially hazardous environmental exposures are ubiquitous, the development of diseases from direct environmental causes is, fortunately, limited. For most diseases, environmental exposures represent one of several factors contributing to the development and progression of disease. Other factors include variation in genetic susceptibility, the presence of other conditions or diseases, diet, activity level, and medications taken. In addition, whether an individual develops disease as a function of environmental exposure also depends on the agent, the extent of exposure, and the timing of exposure. Nevertheless, the complex interaction between these factors accounts for the development of the majority of common diseases in the United States, including asthma, diabetes mellitus, atherosclerosis, and many types of cancer. The question we’d like for you to consider is what tools are needed to understand the role of environmental exposures in the development of common complex diseases.

Traditional methods for human exposure assessment involve measuring exposure concentrations in the ambient environment and extrapolating to potential points of human contact, as well as measuring the concentration of parent compounds or their metabolites in biological samples (biomonitoring). However, both these methods are problematic: measures of the ambient environment represent only rough estimates of an individual’s exposures, and biomonitoring provides only transient estimates of exposure.

We believe the time is right to advance a new science focused on the interface between exposures and human health. Technologies currently exist for the global analysis of genes (genomics), gene transcripts (transcriptomics), proteins (proteomics), and metabolites (metabolomics). Emerging fields, such as medical imaging, nanotechnology, and sensor technology are beginning to yield products that affect the way we conduct research and are proving useful in homeland security and biodefense. Our view is that these technologies also represent important opportunities for providing tools that could advance our understanding of disease etiology by providing quantitative methods to assess the temporal and biological response to multiple environmental exposures. Ideally, these new technologies will generate insight on exposures across the exposure–disease continuum, from the point of human contact to the internal dose to the early biological response (see figure above).

Our vision at the NIEHS is to use environmental health sciences to understand human disease and improve human health. Fundamental to this vision is the ability to quantify an individual’s exposure, as well as the unique characteristics that account for individualized responses to common exposures. To achieve this ability, we must develop an exposure biology initiative that will provide the same degree of individual-level precision that is being achieved through the sequencing of the human genome.

The question to consider is what tools are needed to understand the role of environmental exposures in the development of common complex diseases.

While this will not be easy, the impact of this scientific infrastructure on advancing our understanding of disease etiology and pathogenesis will be profound; in the absence of this infrastructure, it will remain difficult to evaluate the relationship between environmental exposures and human health. To the extent feasible, new technological developments should complement efforts that are ongoing in the public and private sectors, such as biodefense and national health surveillance. Realistically, investments will be needed in long-term research and training programs to enhance the precision and applicability of environmental exposure measurements to human health research.

As part of its strategic planning process, the NIEHS will establish priorities and develop a plan to move exposure health sciences forward. The emphasis will be on advancing our knowledge of gene-environment interactions in model disease processes. Deciphering the environmental and genetic risk factors for disease development and progression, specifically the interaction between environmental exposures and gene differences, will enable researchers and clinicians to develop better strategies for modifying risk and treating disease. Hence, the ability to develop, validate, and correlate exposure-response indicators with allele variation will be critical to our success in reducing the burden of human disease. As always, we look forward to your comments.

## Figures and Tables

**Figure:**
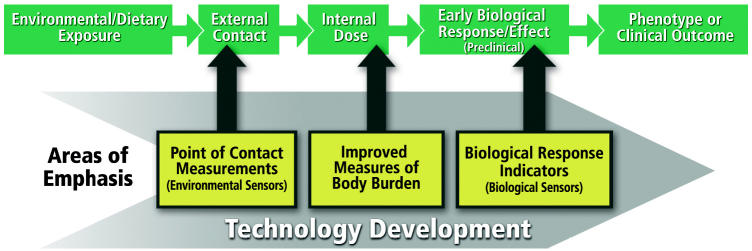


**Figure:**